# Nonlinear plasmonic dispersion and coupling analysis in the symmetric graphene sheets waveguide

**DOI:** 10.1038/srep39309

**Published:** 2016-12-15

**Authors:** Xiangqian Jiang, Haiming Yuan, Xiudong Sun

**Affiliations:** 1 Department of Physics, Harbin Institute of Technology, Harbin 150001, China

## Abstract

We study the nonlinear dispersion and coupling properties of the graphene-bounded dielectric slab waveguide at near-THz/THz frequency range, and then reveal the mechanism of symmetry breaking in nonlinear graphene waveguide. We analyze the influence of field intensity and chemical potential on dispersion relation, and find that the nonlinearity of graphene affects strongly the dispersion relation. As the chemical potential decreases, the dispersion properties change significantly. Antisymmetric and asymmetric branches disappear and only symmetric one remains. A nonlinear coupled mode theory is established to describe the dispersion relations and its variation, which agrees with the numerical results well. Using the nonlinear couple model we reveal the reason of occurrence of asymmetric mode in the nonlinear waveguide.

At THz and far-infrared frequency range, the electrons transition of intraband dominates primarily and the metallic conductivity of Drude type makes the graphene surface plasmon be supported. Based on its unique electric and optical properties^1^ graphene has been suggested as an alternative to conventional metal-based structures to confine light and guide surface plasmon polaritons. Electromagnetic properties of graphene-dielectric composite structures have attracted special attention in the past years, leading to the rapid development of a new branch of plasmonics known as graphene plasmonics^2^,^3^,^4^,^5^.

Considerable effort has been devoted to investigating the mode propagation^6^,^7^,^8^,^9^,^10^, localization^11^,^12^ and coupling^13^,^14^,^15^,^16^,^17^,^18^,^19^,^20^,^21^ of graphene plasmon in the linear graphene-dielectric composite structures. The propagation properties of guided graphene plasmon in individual and paired graphene ribbons were studied^6^, and the features of low loss, large confinement of light and flexible tunability were found. To manipulate the energy flow of light, Wang *et al*.^7^ proposed a graphene plasmonic lens^7^, this lens can be used to focus and collimate the graphene plasmon waves propagating along the graphene sheet. The confinement of plasmon in very small regions has potential applications in optoelectronics, the surface plasmon resonance in graphene sub-nanometre scale has been explored^11^.

The coupling effects of graphene plasmon have attracted wide interest. The demonstration of surface plasmon excitation in graphene based on the near-field scattering of infrared light has been reported^13^,^14^. Recently, Constant *et al*.^15^ presented an all-optical plasmon coupling scheme which takes advantage of the intrinsic nonlinear optical response of graphene, and found that surface plasmons with a defined wavevector and direction can be excited by controlling the phase matching conditions. To realize ultra-high contrast optical modulators, the phase-coupling scheme of localized graphene plasmon resonances has been proposed to replace the original near-field coupling^17^. Moreover, the tunable multiple plasmon induced transparencies based on phase-coupling has been demonstrated by the same group^18^. For the graphene-dielectric multilayer structure, the mode coupling properties and its control are useful for designing compact and tunable nanophotonic devices. It is shown that the graphene-dielectric-graphene waveguide can support both symmetric and antisymmetric modes^19^,^20^. When the graphene sheets are arranged periodicly and tightly, the strong coupling between surface plasmon polaritons emerges^21^.

As was shown, graphene is a strongly nonlinear material^22^,^23^. Several nonlinear optical effects based on graphene’s nonlinearity were predicted^24^,^25^,^26^,^27^,^28^. A novel class of nonlinear self-confined modes originated from the hybridization of surface plasmon polaritons with graphene optical soliton is demonstrated to exist in graphene monolayers^25^. In order to increase the nonlinearity of photonic structures with graphene, the graphene multilayer structure is presented. The nonlinear switching and palsmon soliton based on graphene multilayer were demonstrated^26^,^27^. For the nonlinear graphene-dielectric-graphene structure^26^, the symmetric, antisymmetric and asymmetric mode were found in the structure. The occurrence of asymmetric mode means the symmetry breaking phenomenon. However, the mechanism of symmetry breaking is still unclear although the phenomenon was found in nonlinear plasmonic waveguides. Therefore, the purpose of this article is to study nonlinear plasmonic dispersion and coupling properties in symmetric graphene sheets waveguide, and reveal the mechanism of symmetry breaking phenomenon.

## Results

### Nonlinear modes and dispersion properties

**Figure 1:**
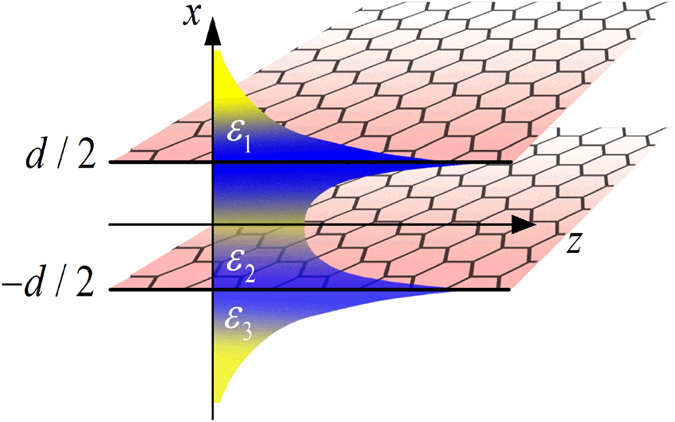
Schematic diagram of nonlinear symmetric graphene sheets plasmonic waveguide, *ε*_1_ = *ε*_3_ = 1, *ε*_2_ = 2.25.

The nonlinear graphene plasmonic waveguide is illustrated in Fig. 1. The dielectric slab waveguide of *ε*_2_ is bounded by the graphene layers at *x* = ±*d*/2 with the surrounding dielectric (*ε*_1_ = *ε*_3_). According to the Kubo formula^29^, the linear conductivity of grapheme *σ*_*L*_ contains the interband and intraband transition contributions. In the THz and far-infrared frequency range, the intraband transition dominates the linear conductivity of graphene which can be reduced to the Drude form^29^


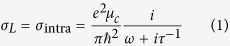


where *e* is the electron charge, *μ*_*c*_ is the chemical potential of graphene, *ω* is the frequency, and *τ* is the momentum relaxation time. This model is applicable in low temperature limit (*k*_*B*_*T *≪ *μ*_*c*_) at low frequency (*ħω *≤ *μ*_*c*_).

For the strong field condition, the nonlinear part of the conductivity must be considered and the total conductivity of graphene reads^27^


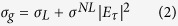


where *E*_*τ*_ is the tangential component of the electric field and *σ*^*NL*^ denotes nonlinear conductivity


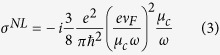


where *ν*_F_ = 0.95 × 10^8^cm/s is the Fermi velocity.

Considering the transverse-magnetic (TM) surface plasmon polaritons mode that propagates along *z* direction with a propagation constant *β*, the magnetic and electric field should be in the form of **H** = *H*_±,*y*_ exp (*iβz* ± *K*_*x*_*x*)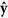 and 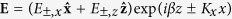 in the dielectrics or air, respectively, where 
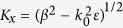
 and *k*_0_ = ω/*c*. According to the boundary condition, the tangential component of electric field must be continuous while that one of the magnetic field has a discontinuity of *σ*_*g*_*E*_1,+,*z*_, i.e.,


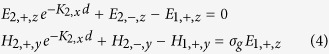


‘±’ in the subscript represents the field decrease and increase along upward direction of *x*. Similar boundary condition was also established at lower boundary. The Maxwell equation gives the relation


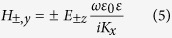


Applying Eq. (5) to region 1, 2 and 3, the dispersion relation equation was obtained with unknown variations of (*β*,*ω*,*H*_1,−*z*_  ≡ *H*_0_).

**Figure 2:**
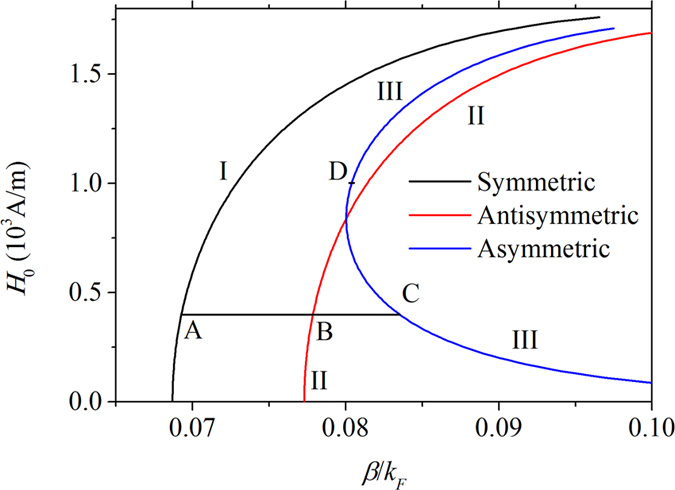
The initial magnetic intensity versus the propagation constant for the fixed wavelength *λ* = 10 μm, the horizontal black solid line is an auxiliary line.

Dependence of the magnetic field *H*_0_ on the propagation constant *β* at wavelength *λ* = 10 μm is shown in Fig. 2, where other parameters are fixed to the values *d* = 100 nm, *ε*_1_ = 1, *ε*_2_ = 2.25, *μ*_*c*_ = 0.27 eV and *τ* = 1.5 ps. The propagation constant *β* is normalized by Fermi vector *k*_F_ = (*πn*)^1/2 ^30 with the carrier density of *n* = 6 × 10^12^ cm^−2^. There are three modes in the nonlinear plasmonic waveguide, which are symmetric mode, antisymmetric mode and asymmetric mode^26^. However, it is impossible to distinguish which branch denotes symmetric, antisymmetric or asymmetric mode. To verify the mode properties of these branches in Fig. 2 we plot electric field and magnetic field distribution associated with A, B, C and D, respectively.

**Figure 3:**
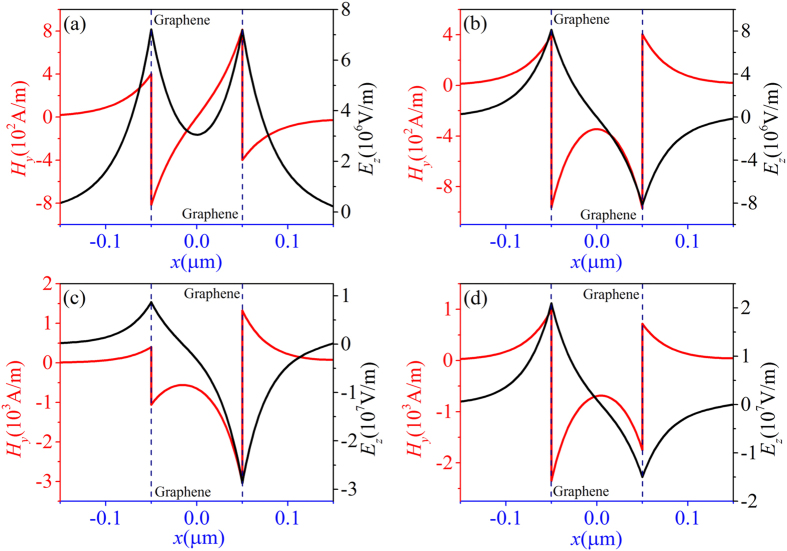
The field distribution for magnetic component *H*_*y*_ and electric component *E*_*z*_, which correspond to points A, B, C and D in Fig. 2, respectively. (**a**) (*H*_0_, *β*) = (400A/m, 0.0779*k*_*F*_), (**b**) (*H*_0,_ *β*) = (400A/m, 0.0693*k*_*F*_), (**c**) (*H*_0_, *β*) = (400A/m, 0.0835*k*_*F*_), (**d**) (*H*_0_, *β*) = (1000A/m, 0.0805*k*_*F*_). Other parameters are the same as in Fig. 2.

For branch I the fields are plotted in Fig. 3(a), in which distribution of electric field *E*_*z*_ is a symmetric. Therefore, the branch I represents the symmetric mode. For branch II distribution of electric field *E*_*z*_ shown in Fig. 3(b) is antisymmetric. It corresponds to the antisymmetric mode. The branches I and II represent symmetric and antisymmetric modes with respect to the linear conditions. They are caused by coupling of graphene plasmon on the upper and the lower air/graphene/dielectric interfaces. Another branch III is a novel mode which appears only due to nonlinearity. It yields to an interesting field distributions associated with C and D at branch III which are plotted in Fig. 3(c) and (d). Corresponding field distribution is asymmetric, and therefore branch III represents asymmetric mode.

**Figure 4:**
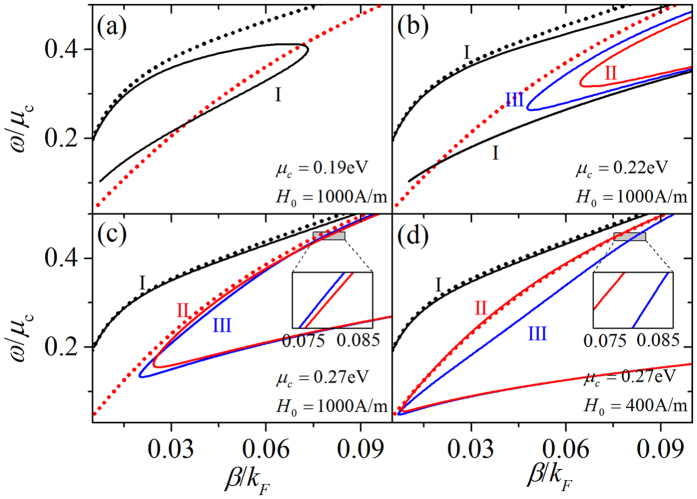
The dispersion relations for various nonlinearity of graphene. The dotted curves represent the dispersion relation of linear case, and the solid curves denote the dispersion relation of nonlinear case. The parameters (*μ*_*c*_, *H*_0_) are chosen to (**a**) (0.19 eV, 1000A/m); (**b**) (0.22 eV, 1000A/m); (**c**) (0.27 eV, 1000A/m); (**d**) (0.27 eV, 400A/m).

Next, we turn our attention to discuss the influence of nonlinearity of graphene on dispersion relation. In Fig. 4, the dispersion relations are depicted with the dotted curves in linear case (*σ*^*NL*^ = 0) and by the solid curves in nonlinear case. For the linear case only symmetric and antisymmetric modes exist. The black dotted curve and the red dotted curve represent the symmetric and antisymmetric modes, respectively. In Fig. 4(a–c) dispersion relation for fixed initial magnetic field (*H*_0_ = 1000 A/m) and different chemical potentials *μ*_*c*_ is given. As is shown in Fig. 4(a), for the larger nonlinearity, when *μ*_*c*_ = 0.19 eV, only symmetric mode represented by the solid curve is found. It is seen from Fig. 4(b) that at chemical potential is equal to 0.22 eV, antisymmetric (red solid curve) and the asymmetric (blue solid curve) modes appear in addition to symmetric mode of branch I. Further increase chemical potentia l (*μ*_*c*_ = 0.27 eV) leads to the intersection of antisymmetric and asymmetric modes, which is seen in Fig. 4(c). In Fig. 4(d), these results are compared to those obtained at constant value of chemical potential *μ*_*c*_ = 0.27 eV, and to decreased initial magnetic field *H*_0_. Decrease of *H*_0_ leads to consequent reduction of nonlinearity of graphene. In this case the fold-back point of the dispersion relations moves down. In addition, as is shown in Fig. 2, there is a intersection of the antisymmetric and asymmetric branch. Therefore, red and blue modes show an opposite trend when the wavelength of the insets in Fig. 4(c) and (d) is about 10 μm (*ω*/*μ*_*c*_ = 0.45). The lower branch of mode I is not plotted in Fig. 4(c) and (d), since it is too close to the lower branch of mode II and III. Nevertheless, it exists.

### Nonlinear coupled mode theory

**Figure 5:**
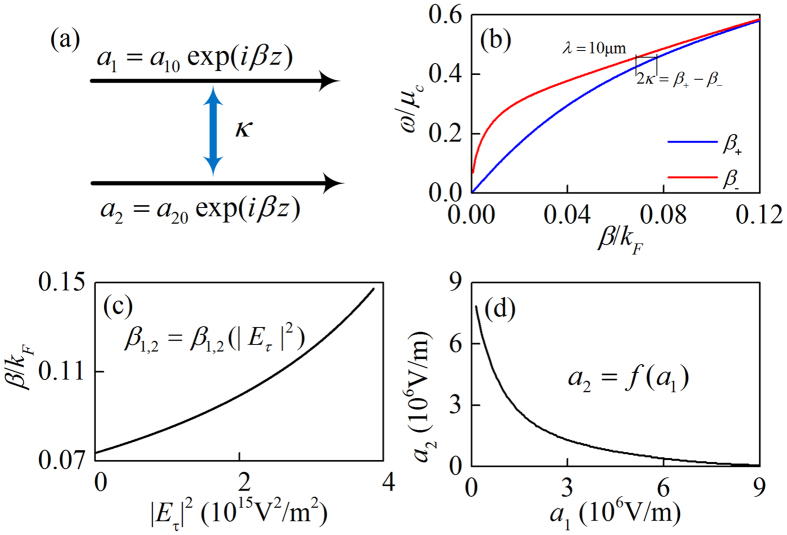
(**a**) The scheme of the coupling between two graphene waveguides. (**b**) The dispersion relations of the linear bi-graphene waveguide with distance *d*. (**c**) The nonlinear induced propagation constant change of a single graphene waveguide. (**d**) The third solution *a*_2_ = *f*(*a*_1_) which satisfying Eq. (10) and Eq. (11).

In the case of weak field without nonlinearity, the coupled graphene plasmonic waveguide shown in Fig. 1 are depicted in Fig. 5(a). According to the coupled mode theory^31^, the oscillation energies *a*_1_ and *a*_2_ satisfy the matrix equation


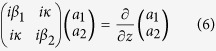


where *β*_1_ and *β*_2_ are the propagation constants of the single layer graphene waveguide without coupling and *κ* is the coupling coefficient. The weak field condition of symmetric structure without nonlinearity corresponds to *β*_1_ = *β*_2_ = *β*_0_. The propagation constants of the coupled mode are defined as the eigenvalues of the matrix


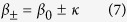


They could also be obtained from the mode analysis method. Figure 5(b) presents the dispersion relations of the same structure as is shown in Fig. 1. In this case the coupling coefficient is found to be *κ* = 4.3 × 10^−3^*k*_*F*_ at wavelength *λ* = 10 μm.

When the graphene’s nonlinearity is considered, the propagation constant of each single graphene waveguide becomes a function of tangential component of electric field. The dispersion of the single graphene waveguide is^32^


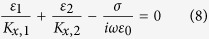


where 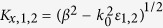. Substituting Eq. (2) into Eq. (8) one gets the nonlinear propagation constant of the single layer graphene waveguide which is shown in Fig. 5(c). Replacing *β*_1_ and *β*_2_ in Eq. (6) with *β*_1,2_(|*E*_*τ*_|^2^) and treating *a*_1,2_ as the tangential component of electrical field in amplitude, we obtain the coupled mode theory in nonlinear case


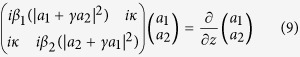


where |*a*_1_ + *γa*_2_|^2^ and |*a*_2_ + *γa*_1_|^2^ are the total field intensity with the similar meaning to the |*E*_1,*τ*,*+*_|^2^ and |*E*_3,*τ*,*−*_|^2^, respectively, and *γ* is an empiric factor related to *β* and *d*, which is fitted from the numerical data shown in Fig. 2. For propagation along the z direction (∂_*z*_ = *iβ*), *a*_1_ and *a*_2_ must satisfy Eq. (10) and Eq. (11) simultaneously


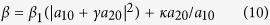



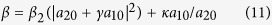


**Figure 6:**
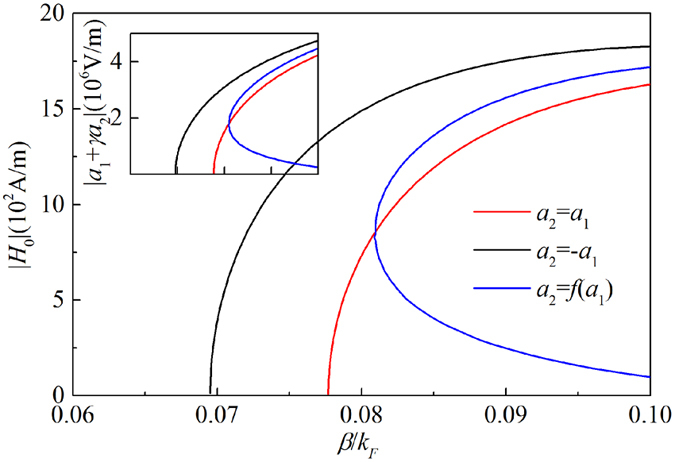
The initial magnetic field *H*_0_ versus propagating constant *β* derived from nonlinear coupled mode theory after the transformation of |*a*_10_ + *γa*_20_| → |*H*_0_|. The inset is *β* vs. |*a*_1_ + *γa*_2_| with *κ* = 4.3 × 10^−3^*k*_F_ and *γ* ~ −0.07.

The first two solutions are *a*_20_ = ±*a*_10_, and the third one can be only obtained numerically shown in Fig. 5(d). Thus, *a*_20_ = ±*a*_10_ and *a*_20_ = *f*(*a*_10_) represent three branches obtained from Eq. (10) (or Equation (11)). The theoretical result from the coupled mode theory in nonlinear case at a proper value of *γ* ~ −0.07is shown in Fig. 6. The relationship |*H*_0_|~|*a*_1_ + *γa*_2_|*ωε*_0_*ε*_1_/*K*_*x*,1_ can be used. It is found that the theoretical result consistent with the numerical one shown in Fig. 2.

The symmetric condition of *a*_20_ = ±*a*_10_ leads to the symmetric increase of *β*_1_ and *β*_2_, hence, equality *β*_1_ = *β*_2_ is always established. Corresponding branches are presented by black and red curves in Fig. 6 (and Fig. 2), i.e., symmetric and antisymmetric field distribution, respectively. For asymmetric condition *a*_10_ > *a*_20_ > 0 (or *a*_20_ > *a*_10_ > 0), the former term in Eq. (10) is larger (smaller) than that one in Eq. (11), but the latter term had an opposite order. When these two variation become equilibrium at *a*_20_ = *f*(*a*_10_), we have the blue branch as shown in Fig. 6. We can conclude that the asymmetric mode come from the equilibrium of the propagation constant (*β*_1_(|*a*_10_ + *γa*_20_|^2^)) increase caused by the nonlinearity and compensation (*κa*_20_/*a*_10_) due to the coupling.

## Discussion

In summary, the coupled and dispersion properties of the graphene-dielectric-graphene structure are studied. The propagation constant is found to increase with the field intensity for both the symmetric and antisymmetric mode, whereas the antisymmetric mode splits off an asymmetric mode. When the nonlinearity of graphene is small (*μ*_*c*_ = 0.27 eV, 0.22 eV), the dispersion relations shows three branches, and there is a fold-back point in each branch. Continuing to increase the nonlinearity of grapheme (decreasing *μ*_*c*_ to 0.19 eV), the fold-back point disappears and there is only one branch corresponding to the symmetric mode. By introducing the nonlinear coupled mode theory, the features of the nonlinear plasmonic waveguide could be understood well. The reason for emergence of asymmetric mode is revealed. It is originated from the equilibrium of the propagation constant increase caused by the nonlinearity and the compensation due to the coupling.

## Additional Information

**How to cite this article:** Jiang, X. *et al*. Nonlinear plasmonic dispersion and coupling analysis in the symmetric graphene sheets waveguide. *Sci. Rep.* **6**, 39309; doi: 10.1038/srep39309 (2016).

**Publisher’s note:** Springer Nature remains neutral with regard to jurisdictional claims in published maps and institutional affiliations.
